# Supplementary material for: Effects of soccer instruction on the executive functions and agility of children in early childhood
Source: PLoS One. 2024 Oct 23;19(10):e0312265. doi: 10.1371/journal.pone.0312265 (PMC11498701; doi:10.1371/journal.pone.0312265)
Supplement: Attachment — Submitted filename: PLOS ONE article Comments.docx [file pone.0312265.s002.docx]

Reviewer Comments on:

**“Effects of soccer instruction on the executive functions and agility of children in early childhood”**

Overall, the study is an interesting idea, but for better readability it needs improvements in writing and improve particularly in the study's methodology and results that will be discussed in the relevant section.

**Abstract:**

The abstract could be better structured to enhance readability.

The abstract does not mention the outcome related to agility, even though it is a central focus of the study.

In the results sentences: Clarify the comparison of pre- and post-test scores within the intervention and control groups, as the current phrasing might confuse readers.

In overall, by improving the clarity, structure, and inclusion of all relevant results, the abstract would be more comprehensive and aligned with the standards of the journal.

**Introduction:**

The introduction provides a comprehensive overview of the relevance of exercise, particularly open-skill sports like soccer, in enhancing executive function and agility in children. Meanwhile, there are some comments for improving clarity and the flow:

The introduction repeats some information, particularly regarding inhibition and the advantages of open-skill sports. Condensing these points would improve readability, e.g., the paragraph discussing the benefits of open-skill sports (line 86-97) could be streamlined by merging similar ideas and avoiding redundancy in discussing inhibition tasks across different studies.

The section discussing the limitations of previous studies in establishing a causal relationship between soccer participation and improved executive function could be more precise (line:64-70 and 79-85). It might help to explicitly state that the lack of baseline measures is a significant limitation in previous research.

The purpose of the study is clear, but it could be emphasized more by separating it into its own paragraph and explicitly stating the research questions or hypotheses.

**Method:**

To report average and mean of the age for five years old, by months or year ±SD, Refer to the PLOS one priority format (line 110- 114).

The methodology is well-structured and covers essential aspects of the study, but it has several weaknesses that could affect the study's validity, reliability, and generalizability. Addressing these issues would support the study design and improve the strength of the findings, e.g.,:

*As a Small Sample Size study*: The intervention group consists of only 31 children, and the control group has 39. While this might be sufficient for a pilot study, the small sample size limits the generalizability of the findings. The results may not be representative of the broader population of five-year-old children. Then it needs to be considered as the study limitation.

*As a Single Study Location:* All participants are from the same kindergarten in the same prefecture, which further limits generalizability. The study’s findings might not apply to children in different geographical locations, cultural settings, or educational environments. If there is not any confidential conflict, it is better to write fully name of kindergarten (line 110 and 113).

It needs to clarify the randomization. Since the decision to participate in soccer instruction was made by the parents, leading to a potential self-selection bias. Then, a randomized controlled trial (RCT) design would be stronger, if children were randomly assigned to either the intervention or control group to minimize selection bias.

To control group activity, it needs to provide a detailed description of the control group’s activities or consider using an active control group that participates in another structured, non-soccer activity.

Blinding the researcher to group allocation during the assessment and analysis phases would support lessen the study bias.

*Outcome Measures:* The study measures inhibition and working memory, but other aspects of executive function, such as cognitive flexibility and planning, are not assessed that can be considered as limitation of the study findings.

*Statistical Analysis:* Lack of Detail on Statistical Methods: Include a more detailed description of the statistical methods used, such as the type of tests (e.g., t-tests, ANOVA, regression analysis), how assumptions were checked, and how missing data were handled. While the methodology mentions using a two-way ANOVA, it’s important to ensure that the assumptions of ANOVA (e.g., homogeneity of variances, normality) are met.

No Mention of Effect Size or Power Analysis: The section does not mention whether effect sizes were calculated or whether a power analysis was conducted to determine the appropriate sample size.

*Ethic:* Include the name of the institution, the approval number, and the date of approval.

**Results:**

The results section provides a solid foundation for presenting the study's findings, but there are several areas where clarity and detail could be improved.

Clarify the interpretation of the interaction effect. Explain what the significant interaction means in the context of the study. For example, discuss whether the intervention was more effective for certain groups or conditions and how this relates to the overall study objectives.

Provide a brief interpretation of the effect sizes, explaining whether they are small, medium, or large according to conventional benchmarks. This will help readers understand the practical significance of the findings. Ensure that the comparison between pre and post-intervention within the control group is clearly stated, without unnecessary repetition or ambiguity

**Discussion**

Discussion is well-structured and provides a comprehensive analysis of the study's findings. However, I have a few suggestions to improve clarity, coherence, and flow:

The discussion touches on the practical implications of the findings, particularly for early childhood education, but these points could be more clearly articulated.

Improvement: Expanding on how these findings could influence the design of physical education programs, or how educators and parents might apply these insights, would strengthen the practical relevance of the study.
